# Building pangenome graphs

**DOI:** 10.1101/2023.04.05.535718

**Published:** 2024-10-02

**Authors:** Erik Garrison, Andrea Guarracino, Simon Heumos, Flavia Villani, Zhigui Bao, Lorenzo Tattini, Jörg Hagmann, Sebastian Vorbrugg, Santiago Marco-Sola, Christian Kubica, David G. Ashbrook, Kaisa Thorell, Rachel L. Rusholme-Pilcher, Gianni Liti, Emilio Rudbeck, Sven Nahnsen, Zuyu Yang, Moses Njagi Mwaniki, Franklin L. Nobrega, Yi Wu, Hao Chen, Joep de Ligt, Peter H. Sudmant, Nicole Soranzo, Vincenza Colonna, Robert W. Williams, Pjotr Prins

## Abstract

Pangenome graphs can represent all variation between multiple reference genomes, but current approaches to build them exclude complex sequences or are based upon a single reference. In response, we developed the PanGenome Graph Builder (PGGB), a pipeline for constructing pangenome graphs without bias or exclusion. PGGB uses all-to-all alignments to build a variation graph in which we can identify variation, measure conservation, detect recombination events, and infer phylogenetic relationships.

## Full Text Availability

The license terms selected by the author(s) for this preprint version do not permit archiving in PMC. The full text is available from the preprint server.

